# Leclercia adecarboxylata in a Patient With Newly Diagnosed Chronic Lymphocytic Leukemia

**DOI:** 10.7759/cureus.57965

**Published:** 2024-04-10

**Authors:** Nivedha Balaji, Aarushi Kalra, Aleksandra Ignatowicz, Tanya Aggarwal, Sonu Gupta

**Affiliations:** 1 Internal Medicine, Northeast Georgia Medical Center Gainsville, Gainesville, USA

**Keywords:** leclericia, immunosuppressed, immunocompromised, lecleria adecarboxylata, l. adecarboxylata

## Abstract

*Leclercia adecarboxylata* is a Gram-negative bacillus commonly seen in immunocompromised individuals and often misdiagnosed as *Escherichia coli*. *L. adecarboxylata* is an opportunistic pathogen found in aquatic environments. It is a nonfatal infection that has low virulence and endorses susceptibility to many common antibiotics. We report a case of a 53-year-old immunocompromised male who was managed for *L. adecarboxylata* bacteremia.

## Introduction

*Leclercia adecarboxylata* is a motile Gram-negative bacillus in the Enterobacteriaceae family known as an opportunistic aquatic organism. It most commonly presents as an acquired infection in the immunocompromised [1, 2]. Due to similar biochemical features, there have been times this microorganism has been misdiagnosed as *Escherichia coli* [3]. However, in the last few years, reported cases of *L. adecarboxylata* have increased in frequency, which may have come from recent outbreaks of product contamination [4]. Bacteremia of this microorganism has been seen in those patients who have skin tears such as burn wounds and cellulitis [5]. Per studies, this low-virulence bacterium has shown high susceptibilities to common antibiotics. In rare cases, sometimes its susceptibility has been limited to first-generation cephalosporin or Trimethoprim (TMP)-Sulfamethoxazole (SMX) due to the resistance of a few strains [6]. In this case report, we discuss the emergence of *L. adecarboxylata* bacteremia in a newly diagnosed immunocompromised patient.

## Case presentation

**Figure 1:**
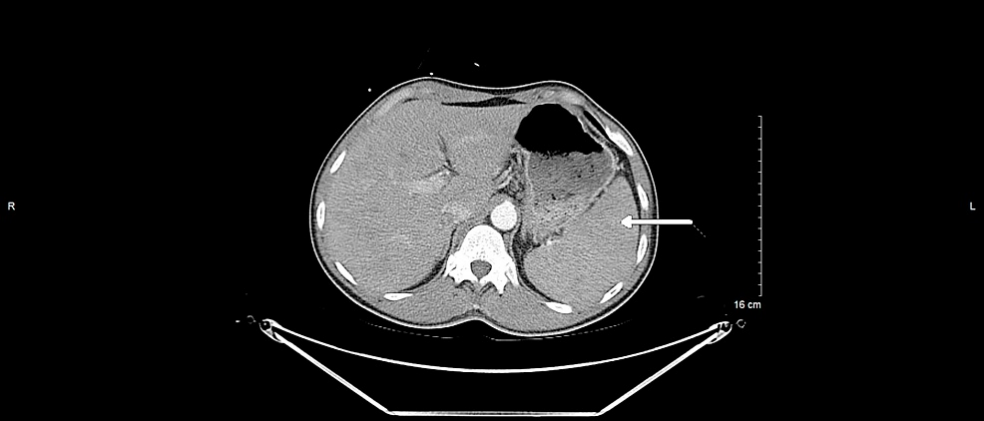
Computed tomography (CT) scan of the chest, abdomen, and pelvis depicting mild splenomegaly. White arrow shows the mild splenomegaly.

**Figure 2:**
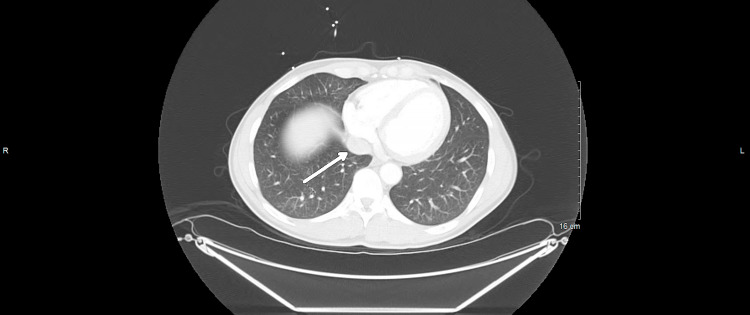
Computed tomography (CT) scan of the chest, abdomen, and pelvis depicting mediastinal lymphadenopathy. White arrow shows the mediastinal lymphadenopathy.

Our case involves a 53-year-old male with no significant medical history who presented to the emergency department with a 5-day history of shortness of breath, malaise, chills, pleuritic chest pain, and productive cough with brown-colored sputum. He endorsed a 20-pound weight loss over a 3-month period and the presence of bilateral inguinal lymphadenopathy of 4-5 weeks duration. Vitals were significant for the temperature of 37.8°C. Laboratory studies were significant for white blood cell (WBC) 42.4 K/μL (5-10 K/μL) with predominant lymphocytosis and procalcitonin 0.40 μg/L (<0.1 μg/L). Physical examination was notable for an ill-appearing male with bilateral inguinal lymphadenopathy without tenderness to palpation. His cardiopulmonary and abdominal exams were unremarkable. Computed tomography (CT) scan of the chest, abdomen, and pelvis shown in Figures 1-2 revealed bilateral ground glass opacities, mediastinal, bilateral hilar, and bilateral axillary adenopathy with mild splenomegaly.

Blood cultures were obtained, and then the patient was started on ampicillin-sulbactam 3 g every 6 hours and azithromycin 500 mg every 24 hours for treatment of community-acquired pneumonia as the CT scan noted bilateral ground glass opacities. Blood cultures grew Enterobacter species *L. adecarboxylata* and *Staphylococcus epidermidis*. *L. adecarboxylata* was pan-sensitive. *S. epidermidis* was deemed a contamination rather than a true infection because it only grew in one bottle. Infectious diseases department was consulted who then initiated amoxicillin 875 mg every 12 hours for an additional 10 days after the patient had completed a 5-day course of ampicillin-sulbactam and azithromycin. With further investigation, the patient reported swimming in a nearby lake several days prior to admission. This is important as the lake may be the source of this patient's infection with *L. adecarboxylata*.

The patient underwent a malignancy workup during the hospitalization due to his presentation with enlarged lymph nodes, elevated WBC count, and due to being infected by *L. adecarboxylata* which is present commonly in the immunocompromised state. Hematology and Oncology were consulted after the leukemia and lymphoma panel was positive for chronic lymphocytic leukemia (CLL). The patient was then discharged and underwent excisional biopsy of the right inguinal lymph node confirming the diagnosis of CLL. Chemotherapy with acalabrutinib and obinutuzumab was initiated at that time. No further follow-up with infectious diseases was required after completion of antibiotic therapy.

## Discussion

In this case presentation, we discuss how *L. adecarboxylata* was diagnosed in a patient who truly fits the risk factor profile for this infection. As mentioned previously, *L. adecarboxylata* is an opportunistic pathogen that is widely present in nature, existing in food, water, soil, and freshwater environments, and is commonly seen in polymicrobial infections [4]. It is also seen in the normal gut flora [2]. It is prevalent in people with predisposing risk factors such as wounds/ulcers, catheters, or immunosuppressive diseases such as cancer, renal failure, or cirrhosis [4, 7, 8]. The morphological and microbiological properties of *L. adecarboxylata* and the Escherichia genus are similar resulting in the misidentification of such infections [7, 8]. After further biochemical testing with DNA hybridization, Leclercia was differentiated from other species in the Enterobacteriaceae family [6].

After the patient stated he was near a body of water prior to hospital arrival and presented with symptoms, sepsis workup labs including respiratory, urine, and blood cultures were collected. His presentation of significant weight loss over the year along with developing lymphadenopathy was concerning for underlying malignancy which prompted further diagnostic testing. The setting where the patient had a biopsy completed during the hospital course showed CLL and blood cultures positive for *L. adecarboxylata*, which only provided further confirmation that this *L. adecarboxylata* was not a contaminant.

Sensitivities of Leclercia were sent and came back pan-sensitive. Many previous cases state that this microorganism is of low virulence [5]. Our patient was initially given cefepime and azithromycin which was transitioned to amoxicillin. A study by Stock et al., showed that 101 strains of Leclercia were naturally susceptible to 70 antimicrobial agents such as tetracyclines, aminoglycosides, most of the beta-lactams, fluoroquinolones, chloramphenicol, nitrofurantoin, and azithromycin; however, they were resistant to penicillin G, oxacillin, erythromycin, roxithromycin, clarithromycin, ketolides, lincosamides, streptogramins, linezolid, glycopeptides, rifampicin, fusidic acid, and fosfomycin [6].

This case portrays the emergence of *L. adecarboxylata* in immunocompromised patients which should not be overlooked. Delay in treatment of *L. adecarboxylata* can lead to complications such as endocarditis, bacteremia, and spontaneous bacterial peritonitis [4, 9]. It can also cause cutaneous diseases such as cellulitis [9, 10]. Early identification and management with antibiotics proves to improve morbidity and mortality.

## Conclusions

In conclusion, this relatively new pathogen has not been studied enough to be considered as a differential on initial presentation for hospitalists, but its recent emergence in immunocompromised patients raises a concern for further evaluation and management. Though susceptible to most antibiotics currently, *L. adecarboxylata*’s resistance can grow over time and may need further studies to discuss worsening complications if management is not sufficient or delayed. This case highlights the need to keep a broad differential when treating patients who are immunocompromised.
